# Impact on Ant Communities by Chemical Pesticides Applied in Controlling the Red Imported Fire Ant (*Solenopsis invicta* Buren) in the Field

**DOI:** 10.3390/insects15110876

**Published:** 2024-11-08

**Authors:** Yunbo Song, Meng Chen, Jiarui Wu, Jingxin Hong, Ting Ouyang, Yuling Liang, Mingrong Liang, Yongyue Lu

**Affiliations:** 1 Red Imported Fire Ant Research Center, South China Agricultural University, Guangzhou 510642, China; songyunbo-scau@stu.scau.edu.cn (Y.S.); 19877989670@163.com (M.C.); wujiarui@stu.scau.edu.cn (J.W.); hongjingxin@stu.scau.edu.cn (J.H.); oyting@stu.scau.edu.cn (T.O.); liangyuling@stu.scau.edu.cn (Y.L.); 2 Insect Biodiversity and Biogeography Laboratory, School of Biological Sciences, The University of Hong Kong, Pok Fu Lam Road, Hong Kong SAR, China

**Keywords:** red imported fire ant, pesticides, reduction, biodiversity, ecological monitoring, ant community

## Abstract

The red imported fire ant (RIFA, *Solenopsis invicta* Buren) represents a significant invasive pest in China, exerting extensive negative impacts on ecosystems. The invasion of RIFA not only poses a severe threat to biodiversity within the environment; inappropriate controlling measures can also adversely affect community dynamics. Therefore, while implementing effective management strategies to control the proliferation of RIFA populations, it is imperative to evaluate the potential effects of these measures on the structure of local biological communities to safeguard native biodiversity. This study employs a “two-step method” using dust and bait formulations, respectively, to control RIFA while conducting ecological monitoring to further assess the impact of RIFA population decline on ant communities. The results of RIFA management showed that after post-treatment periods of 28 days, 35 days, and 60 days, the worker ant reduction rates for the three insecticides—0.5% beta-cypermethrin dust, 1.0% hydramethylnon bait, and 0.1% indoxacarb bait—reached approximately 72%, with their efficacy ranked as follows: 1.0% hydramethylnon bait > 0.1% indoxacarb bait > 0.5% beta-cypermethrin dust. By the 60th day of the experiment, the ant nest reduction rates reached their highest values—66.84% for 0.5% beta-cypermethrin dust, 77.89% for 1.0% hydramethylnon bait, and 87.52% for 0.1% indoxacarb bait—with the latter performing the best. Meanwhile, the occurrence level of RIFAs in all three pesticide treatment areas decreased from level III to level I 60 days post-treatment. Following the application of these three insecticides, the RIFA population significantly decreased, leading to an increase in species richness within the ant community. The reduction in RIFA numbers had a positive impact on the restoration of ant community diversity, as evidenced by significant improvements in both diversity and evenness indices. Notably, 0.1% indoxacarb bait was particularly effective in enhancing the ant community diversity and species richness, while 1.0% hydramethylnon bait was more effective in improving community evenness. These findings indicate that the controlling strategy used in this study not only effectively manages RIFA populations but also promotes recovery and contributes to the ecological balance of local ant communities, providing an important reference for future biodiversity conservation efforts.

## Simple Summary

This study focuses on the management of the red imported fire ant (RIFA), an invasive pest in China that threatens local biodiversity. We implemented a two-step control method using three insecticides, 0.5% beta-cypermethrin dust, 1.0% hydramethylnon bait, and 0.1% indoxacarb bait, and monitored their effects on ant communities. The results showed significant reductions in RIFA populations, with the most effective insecticide being 0.1% indoxacarb bait. After treatment, there was an increase in ant species richness and diversity, indicating a positive impact on local ecosystems. The findings highlight that effective RIFA management can also restore ecological balance among native ant species, providing valuable insights for future biodiversity conservation strategies.

## 1. Introduction

As a globally recognized invasive species, the red imported fire ant (*Solenopsis invicta*) has rapidly spread to multiple regions, including the United States, Mexico, the Caribbean, China, Japan, South Korea, Australia, and Italy [1,2,3,4,5,6,7]. The widespread distribution of the red imported fire ant poses a significant threat to the balance of ecosystems, affecting the predation of arthropods, bird reproduction, and plant growth. Additionally, it causes harm to human health and economic activities [8,9,10,11,12,13]. Therefore, comprehensive quarantine measures and control efforts against the red imported fire ant are crucial to curb its spread. Currently, strategies for managing the red imported fire ant extend beyond chemical control to include biological control and ecological management [6,14]. These strategies not only aid in effectively monitoring and managing the distribution of the ant species but also play a vital role in maintaining ecological balance and ensuring safety in agricultural production.

In field environments, control methods for RIFA can be categorized into three types: physical control, biological control, and chemical control [15]. Among the physical methods, hot water injection has been used as a critical control technique. However, this method is impractical under field conditions and poses risks to surrounding biota. In addition, ant nests may not be eradicated completely by hot water injection, which could potentially lead to the relocation and further spread of RIFA [16,17]. Biological control strategies focus on utilizing parasitic or predatory enemies as well as pathogenic microorganisms to suppress RIFA populations. These approaches aim to control RIFA either by directly eliminating them or indirectly intervening in their competitions with local ant populations [18]. Therefore, biological control may take a certain amount of time for effects to become apparent. In contrast, chemical control is considered to be the most convenient and effective method of rapidly managing RIFA in the short term, primarily involving techniques such as bait toxicity, contact dust treatment, and insecticide mound infusion [19,20,21,22,23,24,25], which have been widely applied in the field. Integrated pest management for RIFA typically employs a two-step method. First, comprehensive monitoring and surveys must be conducted to develop a control plan; this is followed by widespread bait application in high-infestation areas. For localized nests, dust or bait treatments are applied individually, with evaluations of control effectiveness performed two weeks later and targeted management implemented for any still-active nests [18,26,27,28].

While chemical control strategies are widely used for RIFA management due to their efficiency and convenience, their potential ecological impacts should not be neglected. The current study only discusses the effects of pesticides on some specific populations of ants and lacks systematic studies on the recovery of ant community diversity after chemical treatments. The effect of beta-cypermethrin in controlling red imported fire ants under field conditions is notable, as it effectively reduces foraging ant populations [29]. In a study conducted in Orange County, California, hydramethylnon showed significant initial control of red imported fire ants; however, reinfestation occurred in 29–40% of households within three months, underscoring the importance of follow-up treatments to target remaining colonies [30]. Moreover, hydramethylnon not only effectively suppresses the abundance of tropical fire ants (*Solenopsis geminata*) and other invasive ant species, but its application also adversely impacts non-target arthropods, such as cockroaches and crickets [31]. Zakharov et al. [32] further corroborated the effectiveness of hydramethylnon in controlling red imported fire ant populations; however, its efficacy exhibited seasonal rebound patterns and significantly affected non-target ants, particularly those in the subfamily Myrmicinae, potentially leading to local extinctions of these species. Additionally, the use of indoxacarb has also been shown to have negative impacts on non-target predatory true bugs within forest ecosystems [33]. Collectively, these studies emphasize the importance of considering ecological balance in the control of red imported fire ants, reminding us that it is essential to weigh the effectiveness of pesticides against their potential impacts on non-target organisms when developing management strategies.

Therefore, this study not only evaluates the effectiveness of the chemical pesticides 0.5% beta-cypermethrin dust, 1.0% hydramethylnon bait, and 0.1% indoxacarb bait against RIFA but also monitors their impacts on ant communities in the field, further assessing the influence of chemical applications on ant community diversity. To inform future RIFA management strategies, there is a need to strengthen evaluations of ecological impacts and explore more sustainable control methods to achieve both ecological safety and agricultural production security.

## 2. Materials and Methods

### 2.1. Environment and Test Pesticides

This experiment was conducted in an orchard habitat in Huadu district, Guangzhou, Guangdong Province, targeting multi-queen colonies of the red imported fire ants (RIFA). On the day of the experiment, the ambient temperature ranged from 22 °C to 32 °C, with a relative humidity (RH) of 75%. Prior to treatment, the RIFA infestation in both the control and monitoring areas was assessed through visual inspection and baiting methods. For the occurrence level of RIFA, we referred to the Guidelines for Quarantine Surveillance of *Solenopsis invicta* Buren, following the protocol (GB/T 23626-2009) [34], as follows.

1. Classification of Live Ant Nest Density per Unit Area

In regions affected by red imported fire ants, three or more random areas of 500 m^2^ were selected to record the number of live ant nests. The density of live ant nests per unit area was used as the classification criterion, categorized into five levels:

Level I: Mild—an average of 0 to 0.1 live ant nests per 100 m^2^.
Level II: Moderate—an average of 0.11 to 0.5 live ant nests per 100 m^2^.
Level III: Moderately Heavy—an average of 0.51 to 1.0 live ant nests per 100 m^2^.
Level IV: Heavy—an average of 1.1 to 10 live ant nests per 100 m^2^.
Level V: Severe—an average of more than 10 live ant nests per 100 m^2^.

2. Classification of Foraging Worker Ant Density

The number of red imported fire ant workers trapped in monitoring baits was categorized into five levels:

Level I: Light—an average of fewer than 20 workers per monitoring bait.
Level II: Moderate—an average of 20.1 to 100 workers per monitoring bait.
Level III: Moderately Heavy—an average of 100.1 to 150 workers per monitoring bait.
Level IV: Heavy—an average of 150.1 to more than 300 workers per monitoring bait.
Level V: Severe—an average of more than 301 workers per monitoring bait.

When conducting surveys and monitoring using the aforementioned methods, if there is a discrepancy between the density level of live ant nests per unit area and the density level of trapped worker ants, the higher severity level shall be considered authoritative.

**Figure 1:**
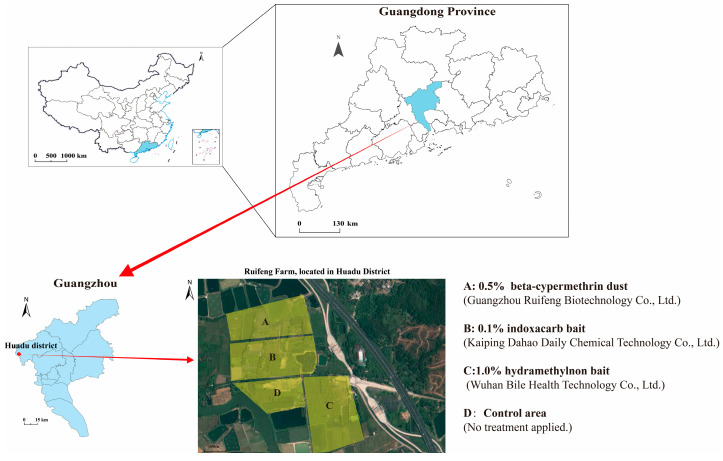
An aerial view of the RIFA control area at Guangzhou Ruifeng plantation. Yellow block A represents the area treated with 0.5% beta-cypermethrin dust (Guangzhou Ruifeng Biotechnology Co., Ltd.), B represents the area treated with 0.1% indoxacarb bait (Kaiping Dahao Daily Chemical Technology Co., Ltd.), C represents the area treated with 1.0% hydramethylnon bait (Wuhan Bile Health Technology Co., Ltd.), and D represents the control area, which received no treatment.

An overview map of the RIFA controlled demonstration area is provided in Figure 1. The selected pesticides used in the experiment were as follows (Figure 1):

1. 0.5% beta-cypermethrin dust (Guangzhou Ruifeng Biotechnology Co., Ltd., Guangzhou, China): the RIFA control area covered approximately 106 mu (1 mu ≈ 666.67 m^2^) and contained 103 active RIFA nests, with an average of 100.5 workers per monitoring bait, classified as level III in infestation severity.
2. 1.0% hydramethylnon bait (Wuhan Bile Health Technology Co., Ltd., Wuhan, China): the demonstration area covered approximately 90 mu, which contains 129 active RIFA nests, yielding an average of 120.33 workers per monitoring bait, also classified as level III in infestation severity.
3. 0.1% indoxacarb bait (Kaiping Dahao Daily Chemical Technology Co., Ltd., Kaiping, China): the demonstration area covered approximately 90 mu and contained 135 active RIFA nests, with an average of 103.65 workers per monitoring bait, classified as level III in infestation severity.
4. The control area covered approximately 50 mu and contained 94 active RIFA nests, with an average of 142.62 workers per monitoring bait, classified as level III in infestation severity.

In this experiment, we employed a “two-step” method. The first comprehensive nest infusion control was conducted in the RIFA control demonstration area on 26 October 2023. On 16 November 2023, a second round of supplementary control and focused monitoring was performed, targeting localized outbreak points of RIFA worker ants detected in the control area. The number of active worker ants and ant nests was recorded 1–2 days before treatment, as well as on days 7, 14, 21, 28, 35, and 60 post-treatment, to assess the effectiveness of the control measures.

### 2.2. Evaluation of the Control Effect of Three Pesticides on RIFAs

In order to assess the effectiveness of the three insecticides in controlling RIFAs, detection surveys were conducted using the following methods.

The ham sausage baiting method (Shuanghui King of Kings, Shuanghui Group) was used as bait [35]. Slices of ham sausages used as bait were placed into transparent plastic bottles with a capacity of 30 mL, laid horizontally on the ground, allowing the mouth of the bottle to be close to the surface. The baits were typically placed during the active ant activity period from 9:00 to 11:00 on sunny days. Four sub-monitoring areas were set up with 10 sampling points spaced 10 m apart in each. After the baits had been in place for about 30 min, the ants inside were collected, taken back to the laboratory, and stored in a −20 °C freezer. Subsequently, the ants were identified by species, and their numbers were recorded.

Active ant nest determination: An active ant nest was defined as one with more than three RIFA worker ants emerging within one minute of inserting an iron or wooden stick into the nest. Observations to determine if the nests were still active were conducted on days 7, 14, 21, 28, 35, and 60 after the application of pesticide. However, in examine the colony control efficacy, it is necessary to excavate and eliminate the existing ant nest. This action may result in the migration of surviving ants, leading to the formation of additional satellite nests. These new nests could complicate our subsequent efforts in trapping foraging worker ants, and recording data on live ant nests. Therefore, we assessed the efficacy of colony control 60 days post-treatment and subsequently calculated the comprehensive control effect. Before the experiment began, all ant nests within each sub-area were marked with small red flags. Subsequently, all new ant nests that appeared were recorded. The appearance of new ant nests indicates either the formation of satellite nests after baiting or the migration of RIFAs from outside the sub-area.

### 2.3. Evaluation Methods for Pesticide Control Efficacy

The evaluation of the pesticide control effect was conducted according to the methods specified in the national standard “Pesticide—Guidelines for the field efficacy trials(2)—Part 149: Insecticides against *Solenopsis invicta* buren GB/T 17980.149-2009” [36].

The formula used to calculate the reduction rate of the worker ants (*PW*) is as follows:

$$PW\left(\%\right)=\left(1-\frac{WO\times WTi}{WOi\times WTO}\right)\times 100$$

*WO*: The average number of worker ants in the monitoring baits in the control area before treatment.
*WTi*: The average number of worker ants in the monitoring baits in the treated area after treatment.
*WOi*: The average number of worker ants in the monitoring baits in the control area after treatment.
*WTO*: The average number of worker ants in the monitoring baits in the treated area before treatment.

The formula for calculating the ant nest control effect (*PN*) is

$$PN\left(\%\right)=\left(1-\frac{NO\times NTi}{NOi\times NTO}\right)\times 100$$

*NO*: The number of active ant nests in the control area before treatment.
*NTi*: The number of active ant nests in the treated area after treatment.
*NOi*: The number of active ant nests in the control area after treatment.
*NTO*: The number of active ant nests in the treated area before treatment.

The formula for calculating the ant colony control effect (*PC*) is

$$PC\left(\%\right)=\left(1-\frac{CO\times CTi}{COi\times CTO}\right)\times 100$$

*CO*: The average level of the ant colonies in the control area before treatment.
*CTi*: The average level of the ant colonies in the treatment area after treatment.
*COi*: The average level of the ant colonies in the control area after treatment.
*CTO*: The average level of the ant colonies in the treatment area before treatment.

The formula for calculating the comprehensive control effect (*P*) of red fire ant is

P=0.3PN+0.2PW+0.5PC

*PN*: The control effect on live ant nests.
*PW*: The control effect on worker ants.
*PC*: The control effect on the ant colony.

### 2.4. Impact of Pesticides on Ant Communities

In order to investigate the effect of insecticides against ant communities, both ham baiting and pitfall trapping were applied to each area, and ant species were recorded before pesticide treatment and 7, 14, 21, 28, 35, and 60 days after treatment.

The pitfall trap method proceeded as follows [37,38]. A plastic centrifuge tube with a length of 115 mm and a diameter of 28 mm was buried underground as a pitfall trap, with the mouth of the tube flushing with the ground surface, and the area around the mouth was filled with soil. The tube contained 1/3 volume of 50% alcohol concentration. Four sub-monitoring areas were set up, with 10 traps placed in each area, arranged in a straight line with approximately 2–3 m between each trap. After the traps were left in place for 24 h, they were collected, and the ants were placed into vials containing 95% alcohol, labeled, and preserved separately. They were then brought back indoors for species identification and counting. After the identification experiment, the ants were placed into vials with 95% alcohol for continued labeling and preservation.

Specimen identification: The collected ants were marked and preserved for identification of species indoors. The identification of ant species mainly refers to the classification systems in “Guangxi Huaping Ant Atlas” [39] and “Common Ants of China Ecological Atlas” [40].

The community characteristic index analysis method proceeded as follows.

(1) Shannon–Wiener species diversity index formula:

$$H^{\prime }=-\sum_{i=1}^{s}\;PiLnPi$$

where Pi = Ni/N, Ni is the number of individuals of the i-th species, and N is the total number of individuals of S species.

(2) Pielou’s uniformity index formula:

$$J=\frac{H’}{lnS}$$

where H′ is the Shannon–Wiener species diversity index and S is the number of species.

(3) Simpson’s dominance index formula calculates the dominance index:

$$D=\sum_{i=1}^{s}{\left(Pi\right)}^{2}=\sum_{i=1}^{s}{\left(\frac{Ni}{N}\right)}^{2}$$

where Pi = Ni /N, Ni is the number of individuals of the i-th species, and N is the total number of individuals of S species [35]. The above data were all processed using Excel.

(4) Margalef’s richness index formula:

$$d=\frac{\left(S-1\right)}{\ln\left(N\right)}$$

where S is the total number of species, and N is the total number of individuals.

### 2.5. Data Analysis

All data analyses were conducted using Microsoft Excel 2013 and Python 3.9.7. Initial data management and preprocessing were performed in Excel, after which the data were imported into Python for further analysis using the pandas library. The scipy.stats module in Python was used to perform a one-way analysis of variance (ANOVA) to assess if any statistical significant differences occurred between the effects of various treatment groups. When significant differences were found using ANOVA, further analyses were conducted using Tukey’s honest significant difference (HSD) test from the statsmodels library to identify specific differences between experimental groups. Finally, data visualization was carried out using Python’s matplotlib and seaborn libraries to generate clear and informative graphs.

## 3. Results

### 3.1. Control Effects of Three Pesticides on RIFA Workers

**Figure 2:**
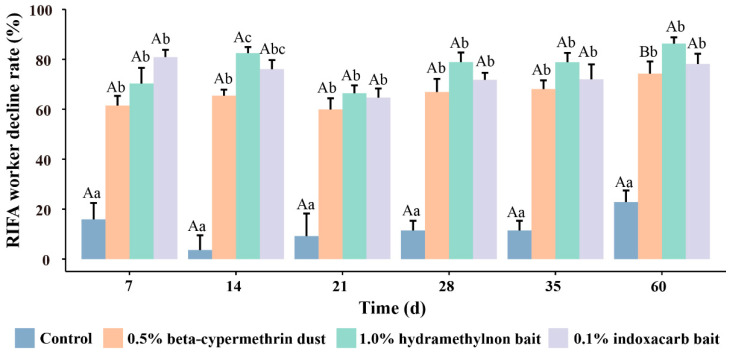
The control effects of three pesticides on RIFA worker ants. The values in the histogram represent the mean ± standard error. Following a one-way analysis of variance (Tukey), identical capital letters indicate no significant difference (*p* > 0.05) between treatments with the same pesticide at different time points. Meanwhile, identical lowercase letters denote no significant difference (*p* > 0.05) between different pesticides at the same time point.

The experimental results indicated that on the seventh day after the start of the treatment, the reduction rates of worker ants for the three pesticides were 61.49% for 0.5% cypermethrin, 70.27% for 1.0% beta-cypermethrin, and 80.88% for 0.1% indoxacarb (Figure 2). These reduction rates were significantly higher than those of the control group (F = 30.818, df = 3, *p* < 0.001). By the 14th day, the reduction rate of 1.0% hydramethylnon bait (82.48%) was significantly higher than that of 0.5% beta-cypermethrin dust (65.44%) and 0.1% indoxacarb bait (76.06%) (F = 87.842, df = 3, *p* < 0.001). On the 21st day, the reduction rates decreased to 59.89% for 0.5% cypermethrin, 66.40% for 1.0% beta-cypermethrin, and 64.67% for 0.1% indoxacarb. A supplementary application of the pesticides was conducted on the 21st day across all experimental groups. On the 28th, 35th, and 60th days, the rates of reduction in worker ants for all three pesticides were stabilized at approximately 72%, with their effectiveness ranked as follows: 1.0% hydramethylnon bait > 0.1% indoxacarb bait > 0.5% beta-cypermethrin dust. Notably, there was no significant difference between the effects of 0.1% indoxacarb bait and 1.0% hydramethylnon bait throughout the treatment period, although 0.5% beta-cypermethrin dust exhibited a relatively higher reduction rate at the 60-day mark (F = 3.71, df = 5, *p* = 0.018).

### 3.2. Control Effects of Three Pesticides on RIFA Nests

**Figure 3:**
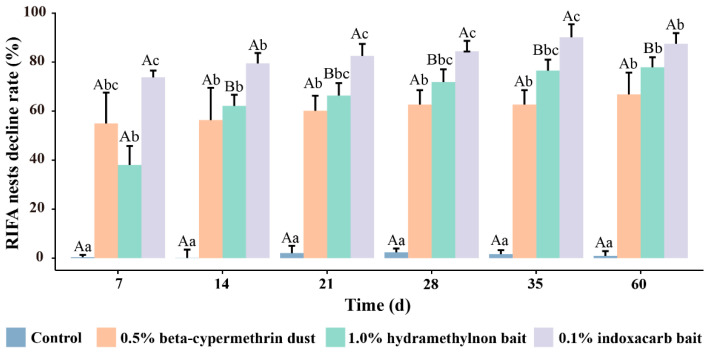
Control effects of three pesticides on *Solenopsis invicta* nests. The values presented in the bar chart are the means ± standard errors. Following a one-way analysis of variance (ANOVA) using the Tukey method, bars with the same uppercase letters indicate no significant difference between treatments with the same pesticide over time (*p* > 0.05). Bars with the same lowercase letters indicate no significant difference between treatments with different pesticides at the same time point (*p* > 0.05).

Seven days after the experiment began, the reduction rates of *Solenopsis invicta* nests were 54.96% for 0.5% beta-cypermethrin dust, 38.01% for 1.0% hydramethylnon bait, and 73.77% for 0.1% indoxacarb bait (Figure 3). The efficacy of 0.1% indoxacarb and 0.5% beta-cypermethrin was significantly higher than that of 1.0% hydramethylnon bait (F = 17.02, df = 3, *p* < 0.01). By the 60th day, the reduction rates peaked at 66.84% for 0.5% beta-cypermethrin, 77.89% for 1.0% hydramethylnon bait, and 87.52% for 0.1% indoxacarb bait. At this stage, 0.1% indoxacarb bait was the most effective, followed by 1.0% hydramethylnon bait, while 0.5% beta-cypermethrin dust was the least effective. During the treatment process, there was no significant difference in the efficacy of the 0.1% indoxacarb bait and the 0.5% beta-cypermethrin dust. Additionally, the reduction rate for the 1.0% hydramethylnon bait on day 14 was significantly higher than on day 7 (F = 126.06, df = 5, *p* < 0.001), indicating a clear improvement in effectiveness over time.

### 3.3. Effects of Three Pesticides on the Control of RIFA Colonies

**Figure 4:**
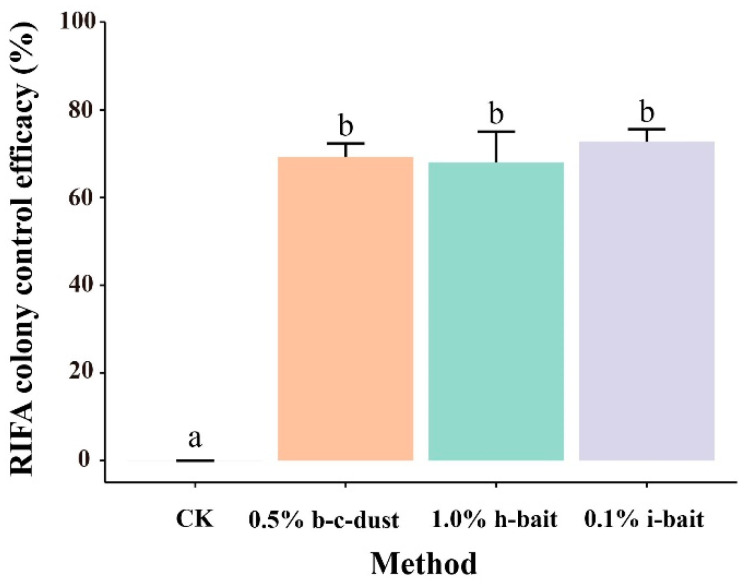
RIFA colony control effects after 60 days of treatment with three pesticides. The values presented in the histogram represent the means ± standard errors. Bars with the same lowercase letters indicate no significant difference between the different pesticide treatments (*p* > 0.05).

The experimental results indicate that the control effects of the three pesticides—0.5% beta-cypermethrin dust, 1.0% hydramethylnon bait, and 0.1% indoxacarb bait—on the RIFA colonies after 60 days were 69.23%, 68.04%, and 72.78%, respectively (Figure 4). Although no significant difference was observed in the control effects among the three pesticides, the treatments were significantly more effective than the control group (*p* < 0.05).

### 3.4. Comprehensive Control Effect of Three Pesticides

**Figure 5:**
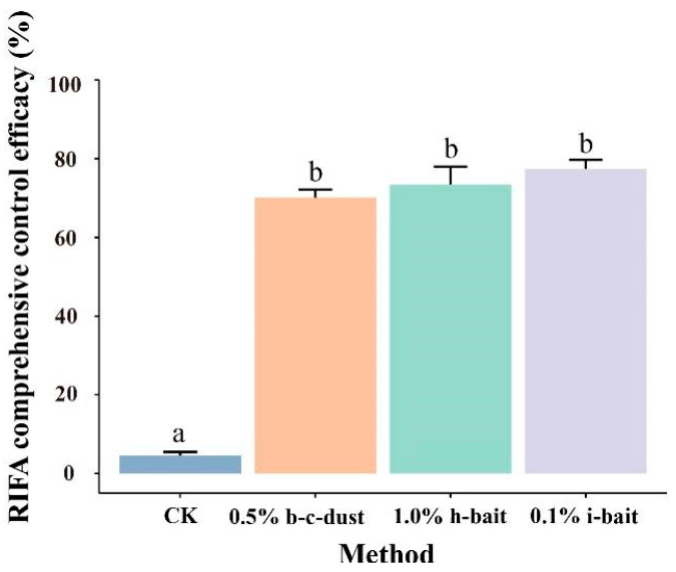
Comprehensive control effects of three pesticides after 60 days of treatment on RIFA. The 0.5% c-dust represents 0.5% beta-cypermethrin dust, 1.0% h-bait represents 1.0% hydramethylnon bait, and 0.1% I-bait represents 0.1% indoxacarb bait. The values presented in the histogram represent the means ± standard errors. The data were analyzed using a one-way analysis of variance (ANOVA) with the Tukey method. Bars with the same lowercase letters indicate no significant difference between the different pesticide treatments (*p* > 0.05).

The experimental results demonstrate that the comprehensive control effects of 0.5% beta-cypermethrin dust, 1.0% hydramethylnon bait, and 0.1% indoxacarb bait on RIFAs after 60 days of treatment were 70.12%, 73.40%, and 77.36%, respectively (Figure 5). Although there were no significant differences in the comprehensive control effects between the three pesticides, all treatments were significantly more effective than the control treatment (*p* < 0.05).

**Figure 6:**
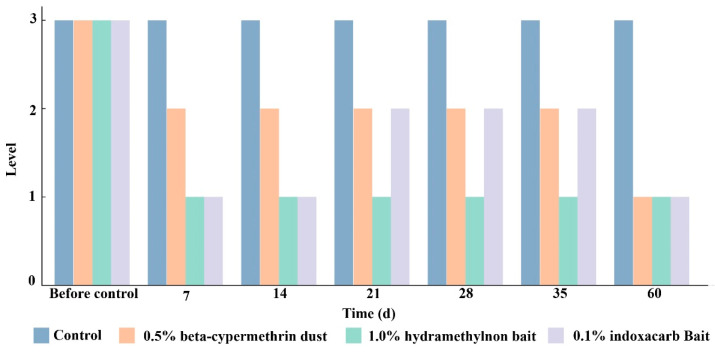
Impact of different treatments on RIFA occurrence levels over a 60-day period. The treatments included a control group, 0.5% beta-cypermethrin dust, 1.0% hydramethylnon bait, and 0.1% indoxacarb bait. According to the quarantine surveillance guidelines for *S. invicta* (GB/T 23626-2009) [34], RIFA occurrence levels were categorized as follows: Level I (Light)—an average of fewer than 20 workers per monitoring bait; Level II (Moderate)—an average of 20.1 to 100 workers per monitoring bait; and Level III (Moderately Heavy)—an average of 100.1 to 150 workers per monitoring bait.

At 7 days post-treatment, the 1.0% hydramethylnon bait and 0.1% indoxacarb bait achieved the most significant reduction in RIFA occurrence levels, while the control remained at level III (Figure 6). The 0.5% beta-cypermethrin dust also reduced levels but was less effective than the hydramethylnon and indoxacarb treatments. Over the 14, 21, 28, 35, and 60-day assessments, the control consistently stayed at level III, showing no reduction. The 1.0% hydramethylnon bait and 0.1% indoxacarb bait maintained lower levels than the control, showing some fluctuations but generally remaining below the control level throughout the period. The 0.5% beta-cypermethrin dust demonstrated moderate efficacy but did not achieve reductions as substantial as the other two baits. After 60 days of management, the occurrence level of RIFA in all three treatment areas decreased from III to I, while the control group remained at level III.

### 3.5. Effects of Three Pesticides on Ant Species

This study assessed the impact of 0.5% beta-cypermethrin dust, 1.0% hydramethylnon bait, and 0.1% indoxacarb bait on the structure of ant communities by applying these pesticides to specific areas and comparing them to untreated control areas. The collected ant samples were classified into three subfamilies, four genera, and four species, including *Tapinoma melanocephalum*, *Paratrechina longicornis*, *Polyrhachis dives*, and *Solenopsis invicta*, as shown in Table 1. The results indicated that a richer variety of ant species were collected from the areas treated with the three pesticides compared to the control area, where only *S. invicta* was found.

**Figure 7:**
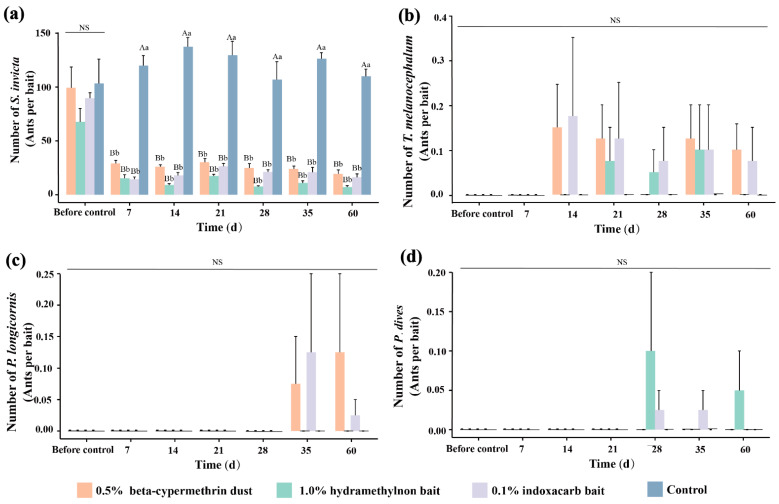
Dynamic changes in ant species (baiting trap). (**a**) Number of *Solenopsis invicta*, (**b**) Number of *Tapinoma melanocephalum*, (**c**) Number of *Paratrechina longicornis*, (**d**) Number of *Polyrhachis dives*. The data in the figure represent the means ± standard errors. Following a one-way ANOVA (Tukey’s method), bars with the same uppercase letters indicate no significant difference among treatments with the same pesticide over time (*p* > 0.05), while bars with the same lowercase letters and “NS” indicate no significant difference among treatments with different pesticides at the same time (*p* > 0.05).

Figure 7 presents the changes in the number of ants collected using the baiting method after treatment with the three pesticides. Before treatment, there was no significant difference between the treated areas and the control area in the number of ants collected (F = 0.957, df = 3, *p* = 0.44). The numbers of *S. invicta* workers collected per bait before treatment were 99.53, 67.73, 89.65, and 103.33. However, during the 7–60-day period following treatments, there were significant differences between the control area and the treated areas (7 days: F = 92.10, df = 3, *p* = 0.00; 14 days: F = 174.63, df = 3, *p* = 0.00; 21 days: F = 58.72, df = 3, *p* = 0.00; 28 days: F = 27.51, df = 3, *p* = 0.00; 35 days: F = 189.9, df = 3, *p* = 0.00; 60 days: F = 137.93, df = 3, *p* = 0.00).

Significant differences were also observed within the treated areas when comparing the number of worker ants collected before treatment and during the 7~ 60-day treatment period. These differences were found for the areas treated with 0.5% beta-cypermethrin dust (F = 12.82, df = 6, *p* = 0.00), 1.0% hydramethylnon bait (F = 19.10, df = 6, *p* = 0.00), and 0.1% indoxacarb bait (F = 63.59, df = 6, *p* = 0.00), whereas no significant differences were observed in the control area.

Additionally, three other ant species were collected in the treated areas after pesticide applications (Table 1 and Figure 7): *T. melanocephalum*, *P. longicornis*, and *P. dives*. *T. melanocephalum* was collected in the area treated with 0.5% beta-cypermethrin dust on days 14, 21, 35, and 60; in the area treated with 1.0% hydramethylnon bait on days 21, 28, and 35; and in the area treated with 0.1% indoxacarb bait on days 14, 21, 28, 35, and 60. *P. longicornis* was collected in both the areas treated with 0.5% beta-cypermethrin dust and 0.1% indoxacarb bait on days 35 and 60. *P. dives* was found in the areas treated with 1.0% hydramethylnon bait and 0.1% indoxacarb bait, appearing on days 28 and 60 in the area treated with 1.0% hydramethylnon bait and on days 28 and 35 in the area treated with 0.1% indoxacarb bait.

**Figure 8:**
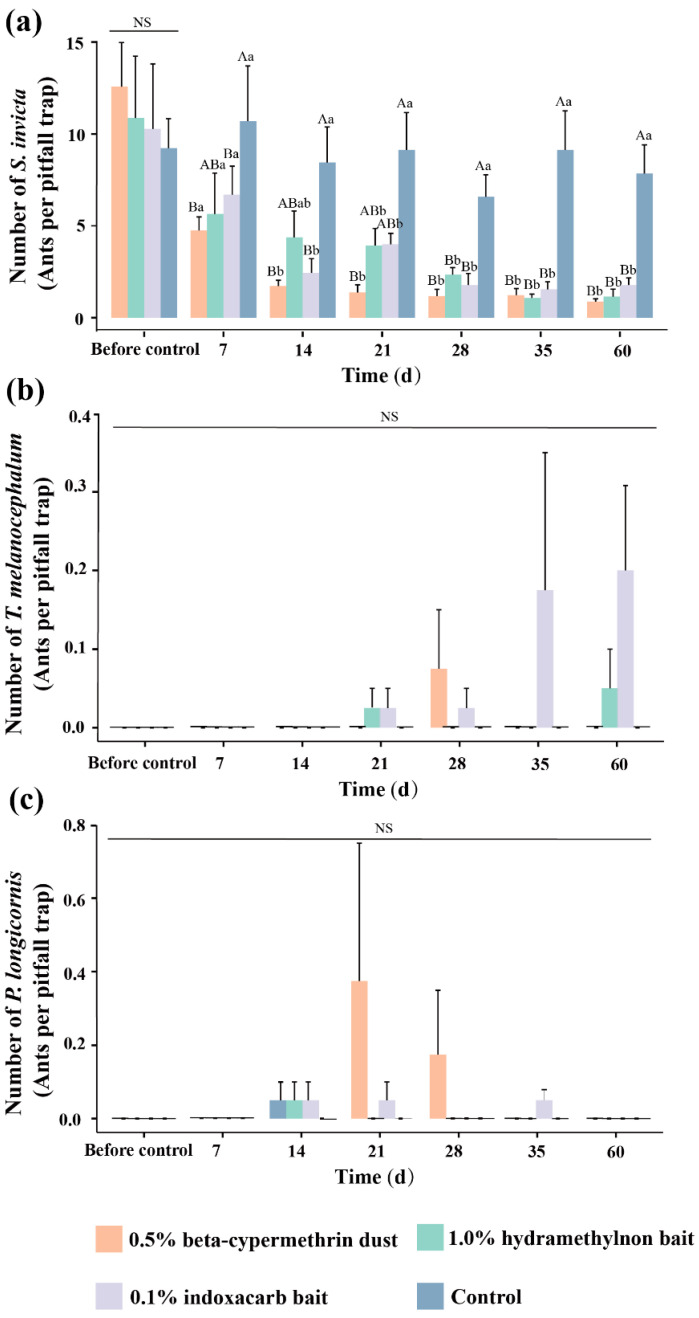
Dynamic changes in ant species (pitfall trap). (**a**) Number of *Solenopsis invicta*, (**b**) Number of *Tapinoma melanocephalum*, (**c**) Number of *Paratrechina longicornis*. The data in the figure represent the means ± standard errors. Following a one-way ANOVA (Tukey’s method), bars with the same uppercase letters indicate no significant difference among treatments with the same pesticide over time (*p* > 0.05), while bars with the same lowercase letters and “NS” indicate no significant difference among treatments with different pesticides at the same time (*p* > 0.05).

After treatment with the three pesticides, the changes in the number of ants collected using the pitfall trapping method are shown in Figure 8. Before the treatment, there were no significant differences between the control area and the areas treated with 0.5% beta-cypermethrin dust, 1.0% hydramethylnon bait, or 0.1% indoxacarb bait (F = 0.25, df = 3, *p* = 0.86). The numbers of RIFA workers collected before treatment were 12.58, 10.86, 10.2, and 9.23 per bait. After 7 days of treatment, there was still no significant difference between the control area and the treated areas (F = 1.63, df = 3, *p* = 0.23).

However, by 14 days post-treatment, significant differences were observed between the areas treated with 0.5% beta-cypermethrin dust and 0.1% indoxacarb bait compared to the control area (F = 5.64, df = 3, *p* = 0.012), while the 1.0% hydramethylnon bait showed no significant difference. From day 21 to 60 after treatment, significant differences in the number of worker ants collected were found between the control area and the areas treated with 0.5% beta-cypermethrin dust, 1.0% hydramethylnon bait, and 0.1% indoxacarb bait (21 days: F = 7.59, df = 3, *p* = 0.04; 28 days: F = 11.21, df = 3, *p* = 0.001; 35 days: F = 12.63, df = 3, *p* = 0.001; 60 days: F = 15.83, df = 3, *p* = 0.00).

Significant differences in the number of workers collected before and after treatment were observed in areas treated with 0.5% beta-cypermethrin dust and 0.1% indoxacarb bait (0.5% beta-cypermethrin dust: F = 18.54, df = 6, *p* = 0.00; 0.1% indoxacarb bait: F = 4.57, df = 6, *p* = 0.04). In the area treated with 1.0% hydramethylnon bait, significant differences in the number of workers were found between pre-treatment and the period of 28 to 60 days after treatment (F = 4.15, df = 6, *p* = 0.07), while there were no significant differences in the number of workers collected during the period from 7 to 21 days after treatment.

Using the trapping method, we collected two additional ant species: *T. melanocephalum* and *P. longicornis*. *T. melanocephalum* was found in the area treated with 0.5% beta-cypermethrin dust at 28 days post-treatment, in the area treated with 1.0% hydramethylnon bait at 21 and 60 days, and in the area treated with 0.1% indoxacarb bait at 21, 28, 35, and 60 days post-treatment. *P. longicornis* was found in the area treated with 0.5% beta-cypermethrin dust at 14, 21, and 28 days post-treatment; in the area treated with 1.0% hydramethylnon bait at 14 days; and in the area treated with 0.1% indoxacarb bait at 14, 21, and 28 days post-treatment.

### 3.6. Change Incommunity Characteristic Indices

**Figure 9:**
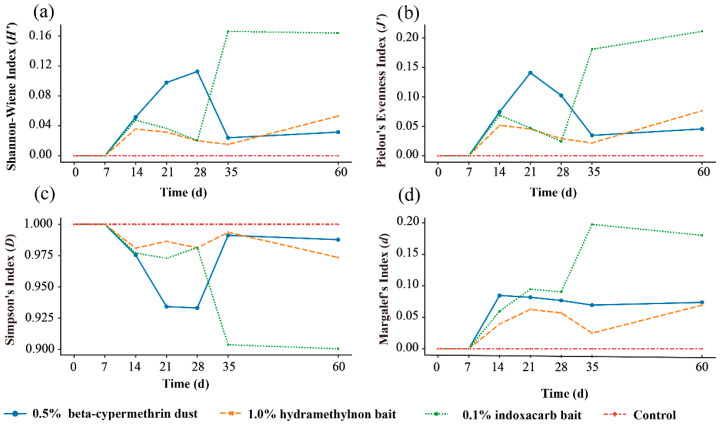
Pattern of characteristic indices changes in the ant community’s response to different pesticide treatments. The data in the chart are the average values of the (**a**) Shannon–Wiener (*H’*) species diversity index, (**b**) Pielou’s (*J’*) evenness index, (**c**) Simpson’s (*D*) dominance index, and (**d**) Margalef’s (*d*) richness index.

After surveying ant communities in areas treated with the three pesticides and in the untreated control area, the Shannon–Wiener (H’) diversity index, Pielou’s (J) evenness index, Simpson’s (D) dominance index, and Margalef’s (d) richness index were measured at different times (Figure 9). The results show that before and 7 days after pesticide treatment, the diversity, evenness, and richness indices in all treatment areas were 0. Throughout the controlling process, the diversity, evenness, and richness indices in the pesticide-treated areas displayed an upward–decline–upward trend. The dominance index was 1 before and 7 days after the pesticide treatment.

The Shannon–Wiener (H’) diversity index results (Figure 9a) indicate that 35 days after treatment, the diversity index in the area treated with 0.1% indoxacarb bait was 0.17, which was significantly higher than in the untreated control area (F = 3.248, df = 8, *p* = 0.23). The diversity index in the area treated with 0.5% beta-cypermethrin dust peaked 28 days after treatment at 0.11, while the diversity index in the area treated with 1.0% hydramethylnon bait reached its highest value of 0.05 at 60 days after treatment.

Pielou’s (J) evenness index for the ant community (Figure 9b) 14 days after treatment showed that the evenness indices for the areas treated with 0.5% beta-cypermethrin dust, 1.0% hydramethylnon bait, and 0.1% indoxacarb bait were 0.075, 0.0517, and 0.069, respectively. At 60 days after treatment, the evenness indices were 0.46, 0.77, and 0.21, respectively. The evenness index in the area treated with 0.5% beta-cypermethrin dust peaked at 0.14, 21 days after treatment, while the indices for areas treated with 1.0% hydramethylnon bait and 0.1% indoxacarb bait reached their highest values at 60 days. Throughout the controlling process, the evenness indices in all three treated areas were higher than those before treatment and in the untreated control area.

The Simpson’s (D) dominance index results (Figure 9c) indicate that after treatment, the dominance index in all regions began to decline, but the red imported fire ant (*Solenopsis invicta*) remained the dominant species in all communities. The dominance index reached its lowest values at 60 days after treatment in areas treated with 1.0% hydramethylnon bait and 0.1% indoxacarb bait, at 0.97 and 0.90, respectively. In the area treated with 0.5% beta-cypermethrin dust, the dominance index reached its lowest value of 0.93 at 28 days.

Margalef’s (d) richness index results (Figure 9d) show that 35 days after treatment, the area treated with 0.1% indoxacarb bait had a richness index of 0.20, which was significantly higher than in the untreated control area (F = 3.721, df = 3, *p* = 0.23). The area treated with 0.5% beta-cypermethrin dust had the highest richness indices at 14 and 60 days after treatment, at 0.08 and 0.07, respectively. The area treated with 1.0% hydramethylnon bait had its highest richness indices 21 and 60 days after treatment, at 0.06 and 0.07, respectively.

These results indicated that pesticide treatment affected ant communities and reduced the dominance of RIFA as the diversity, evenness, and richness indices increased, while the dominance index decreased.

## 4. Discussion

The management and control of the red imported fire ant focus not only on safeguarding human safety and agricultural economies but also on minimizing ecological impacts [41,42,43,44,45]. In agricultural and village habitats, the application of three treatments with 0.05% indoxacarb bait achieved control rates of approximately 65.0% and 66.2%, respectively [46]. In contrast, a single application of 0.1% indoxacarb bait demonstrated a control efficacy of 73.77% after just seven days. Upon employing a two-step method for secondary treatment of surviving colonies 21 days after the initial treatment, efficacy improved to 87.52% by day 60. Similarly, a 1.0% beta-cypermethrin treatment reached 77% efficacy within the same time frame. Additionally, Chen et al. [47] reported that after seven days, 0.1% indoxacarb and 1.0% beta-cypermethrin achieved average control efficacies of 73.13% and 68.16%, respectively. With supplementary applications, the average control rates increased to 95.74% and 94.64%. Following a third application, these rates further rose to 98.85% and 97.97%. Zhou [46] proposed a “four-step ant extermination method”, which yielded control efficacies of 95.9% and 94.1% in both experimental and practical applications, while similar two-step methods with the same pesticides demonstrated over 70% efficacy after 60 days. The RIFA not only competes for resources but also disrupts local ant populations, significantly impacting the diversity, evenness, and dominance of native ant communities. This competition can lead to the local replacement of ant populations by invasive species, threatening ecosystem stability [48,49,50,51]. The invasion of non-native ants results in a decline in local ant species richness, with only a few species able to withstand the invasion [52]. For instance, Lu et al. [53] indicated that the richness of ant species in areas impacted by RIFA decreased by 46% and 33%, respectively. However, a reduction in RIFA populations can facilitate the recovery of local ant diversity, showcasing the ecosystem’s capacity for self-restoration [54]. Moreover, Eubanks et al. [55] observed a significant increase in beneficial arthropod density following RIFA control, underscoring the negative impact of RIFA on arthropod diversity.

The results of the study reveal that after 60 days of management with 0.5% beta-cypermethrin dust, 1.0% hydramethylnon bait, and 0.1% indoxacarb bait, the occurrence level of red imported fire ants in all three treatment areas decreased from level III to level I, effectively reducing worker ant populations by approximately 72%. Among these, 1.0% beta-cypermethrin demonstrated superior efficacy by day 14 compared to the other two baits, while 0.1% indoxacarb yielded optimal results by day 60. Regarding the control effect of the three pesticides on RIFA workers, it can be observed that the reduction rate of worker ants in the control group also increased with time, which was potentially due to the lowering of temperature. However, the data on the combined control effect showed that the control effect in the treated group was still significantly higher than that in the control group. This suggests that our controlling measures played a significant role in the treated group despite the external environmental factors affecting the number of worker ants in the control group. Overall, all three insecticides achieved control efficacies exceeding 70%, with no significant differences noted among the treatments, highlighting their effectiveness in reducing RIFA populations. The effective suppression of RIFA positively influences ant community structure, enhancing species richness and altering community dynamics. Specifically, both 0.1% indoxacarb and 1.0% beta-cypermethrin exhibited promising sustained effects in facilitating the recovery of other ant populations during RIFA control. This observation supports findings by Chen [54], which indicated that successful RIFA management can lead to the recovery and enhancement of other ant communities. In their research, Song et al. [35] explored various application methods and found that both bait and dust significantly decreased RIFA populations, resulting in notable increases in species diversity and evenness. Liu et al. [51] reported varied impacts from different controlling methods on local ant communities, highlighting a post-infusion diversity index of 1.94, which was significantly higher than the pre-treatment index of 1.56. Additionally, Liang et al. [56] investigated plant-based pesticides for RIFA management and observed a reduction from 644 ants to 200 after 14 days, marking a decline of 68.94%, along with significant improvements in diversity and evenness indices post-treatment.

The species richness of native ants is negatively correlated with RIFA density, as RIFA demonstrates a greater competitive ability in contests for food resources [57,58,59]. Gibbons et al. [58] highlighted that RIFA outcompetes several native ant species for resources. Furthermore, habitat disturbances tend to favor RIFA reproduction while simultaneously diminishing native ant populations [60]. The proliferation of RIFA in disturbed environments suggests new avenues for controlling methods.

It is noteworthy that in the treatment area with 1.0% beta-cypermethrin, both baiting traps and pitfall traps did not trap any *P. dives* specimens from the start of the experiment until day 60 (Table 1, Figure 6 and Figure 7). This observation raises the possibility that 1.0% beta-cypermethrin may inhibit the recovery of *P. dives* in the environment; however, it is also conceivable that *P. dives* was potentially absent from this treatment area. These hypotheses require further experiments for future verification.

Additionally, the different recovery times of various ant species may have implications for the detection of ant community diversity metrics, indicating that further experimental setups are necessary to explore these dynamics. The relatively short duration of the ant diversity surveys in this study prompts plans for future research to extend the survey period for a more comprehensive analysis. While baits and dust have proven effective in reducing RIFA populations, they may also impact native ant communities, thus potentially influencing the accuracy of subsequent data. Future investigations will prioritize precise controlling methods to better assess the impact of RIFA management on ant community structures and will also need to monitor weather conditions prior to RIFA worker trapping to avoid the potential impacts of drastic weather changes on the number of foraging workers trapped.

Interestingly, after the RIFA colonies were suppressed in the present study, other ant species recolonized the treated areas, highlighting the interactions among species within the ecosystem. Notably, three out of the four recolonizing ant species (*T. melanocephalum*, *P. longicornis*, and *S. invicta*) are non-native, indicating that although biodiversity and complexity are increasing, the new community is still predominantly composed of non-native species. While *S. invicta* is regarded as one of the most invasive species, its removal does not guarantee the restoration of native diversity; rather, it may lead to a transition toward a more diverse community still dominated by non-native species. Furthermore, invasive alien ants tend to first invade highly degraded habitats and may exacerbate the degradation of these habitats, shedding light on the need to consider broader ecological impacts when managing invasive species.

## 5. Conclusions

Our field trials exhibited that three pesticides—0.5% beta-cypermethrin dust, 1.0% hydramethylnon bait, and 0.1% indoxacarb bait—demonstrated varying reduction rates in worker ants at early stages, achieving an approximately 72% decrease by day 60. The 1.0% hydramethylnon bait was particularly effective by day 14, while the 0.1% indoxacarb bait showed superior performance overall. After 60 days of management, the occurrence level of red imported fire ants in all three treatment areas decreased from III to I. All three treatments significantly reduced RIFA populations, increased ant community species richness, and altered community structure, allowing for the recovery of other species like *T. melanocephalum* and *Polyrhachis dives*. After 35 days of treatment with 0.1% indoxacarb, diversity and richness increased, but by day 60, an uptick in RIFA populations reduced community diversity. Indoxacarb bait effectively diminished RIFA numbers, enhancing the habitat for other ant species, while beta-cypermethrin treatment led to a more homogeneous ant community, suggesting a balanced species proportion.

## Figures and Tables

**Table 1:** Ant species trapped in different pesticide treatment areas (total for both methods).

Species	Indigenous Species/Alien Species	Control Area	Treatment Zones		
			0.5% Beta-Cypermethrin Dust	1.0% Hydramethylnon Bait	0.1% Indoxacarb Bait
*Tapinoma melanocephalum*(Fabricius)	Alien species	-	**+**	**+**	**+**
*Polyrhachis dives* (Smith)	Indigenous species	-	-	**+**	**+**
*Paratrechina longicornis* (Latreille)	Alien species	-	**+**	**+**	**+**
*Solenopsis invicta* (Buren)	Alien species	**+**	**+**	**+**	**+**
Total		1	3	4	4

Note: “ + “ indicates the presence of the ant species in that habitat, and “-“ indicates absence.

## Data Availability

The raw data and materials will be made available by the authors, without undue reservation, to any qualified researchers.
